# Analysis and 3D correction of glenoid dysplasia with metal hemi-wedge base plate augment: short-term radiographic outcomes

**DOI:** 10.1007/s00402-023-04781-6

**Published:** 2023-01-27

**Authors:** Thorsten Guehring, Luis Navas, Jan Westrich, Alexander Zimmerer, Sebastian Schmidt, Miguel Barrientos, Benjamin Ulmar

**Affiliations:** 1 Department of Orthopaedic and Trauma Surgery, Orthopädische Klinik Paulinenhilfe, Diakonie Klinikum, Rosenbergstrasse 38, 70176 Stuttgart, Germany; 2 ARCUS Sportklinik, Rastatterstraße 17-19, 72175 Pforzheim, Germany; 3 Department of Orthopaedics and Orthopaedic Surgery, University Medicine Greifswald, Ferdinand-Sauerbruch-Straße, 17475 Greifswald, Germany; 4 Arthroplasty Division, Mount Sinai Hospital, University of Toronto, 600 University Drive, Toronto, Canada

**Keywords:** Glenoid base plate, Glenoid version, Glenoid inclination, Scapular notching, Preoperative planning, Reversed shoulder arthroplasty

## Abstract

**Background:**

Glenoid defects can be addressed traditionally by asymmetric reaming or by bone-preserving correction to a more lateral joint line by bone or metal augmented baseplates in reverse shoulder arthroplasties. While there is more evidence in literature regarding the outcome and complications of Bony Increased Offset Reversed Shoulder Arthroplasty (BIO-RSA), there is minimal reported experience with the outcome after metal glenoid augments. The aim of this study was to determine whether a metal augment can correct the glenoid deformity in an anatomic manner.

**Methods:**

Glenoid morphology and deformity were determined in 50 patients with Walch type B1, B2, D and Favard type E0–E3 glenoid defects using preoperative radiographic and computed tomography (CT) analysis. All patients received a preoperative planning CT with 3D planning, and measurements of glenoid inclination (in 3 planes proximal, middle, distal), reversed shoulder arthroplasty angle (RSA) and glenoid version were obtained. All patients had a pathologic inclination in the coronal or frontal planes of > 10°. Above the threshold of 10° pathological glenoid version or inclination metal hemi-augments of 10°, 20°, or 30° were used which allow an individual 360° augment positioning according to the patient glenoid deformity.

**Results:**

The mean preoperative numbers of the glenoid version demonstrate that most glenoids were in retroversion and superior inclination. In total 2410° wedges, 1820° wedges and 8 30° wedges were used. In the majority of cases, the wedge was positioned posteriorly and/or cranially between 10:00 and 12:00 o’clock, which allows a correction in a 3D manner of the glenoid inclination and version. The mean RSA angle could be corrected from 22.76 ± 6.06 to 0.19° ± 2.7 (*p* < 0.0001). The highest retroversion of the glenoid is evidenced in the proximal section and it could be corrected from − 23.32° ± 4.56 to − 6.74° ± 7.75 (*p* < 0.0001) and in the middle section from − 18.93° ± 3.35 to − 7.66° ± 5.28 (*p* < 0.0001). A mean sphere bone overhang distance (SBOD) of 5.70 ± 2.04 mm was found in order to avoid or minimize relevant scapular notching.

**Conclusion:**

By using a new 360° metal-augmented baseplate, the preoperative pathological inclination and retroversion can be corrected without medialization of the joint line. Future clinical results will show whether this bone-preserving procedure improves also the clinical outcomes as compared to asymmetric medialized reaming or wedged BIO-RSA.

**Level of evidence:**

Level IV, Case series.

## Introduction

Glenoid defects and glenoid dysplasia can be addressed and corrected by bone or metal augmentation, in order to achieve an effective and accurate lateral and distal prosthetic center of rotation in reversed shoulder arthroplasty. Patients with rotator cuff arthropathy, rheumatoid arthritis of the shoulder, and revision surgery of an anatomic or RSA represent a possibility to preserve shoulder joint function despite missing of rotator cuff [1, 2].

To overcome the weakness or lack of rotator cuff muscles, Grammont stated two important biomechanical principles: (1) medialization of the center of rotation and (2) lowering the humerus [1, 2]. These principles increased the tension on the deltoid muscle, which improves the mechanical advantage on the shoulder elevation, and at the same time reducing the torque on the glenoid component.

An important aspect to be considered is that glenoid defects affect the center of rotation of the shoulder, since the implantation of the baseplate could ream a large amount of bone and therefore the center of rotation could be medialized. In bone defects such as Walch type B or C glenoid, a asymmetric reaming with medialized center of rotation and results in more bone loss but stable sitting of a standard baseplate. This technique has proven to be an alternative without correction of deformity can also lead to good midterm results as shown by Mc Farland et al. However, the detailed amount of deformity corrections remained unclear and some violation of subchondral bone may occur [3].

On the other hand, studies have shown that medialization of the center of rotation may result in multiple complications, for instance, scapula notching, reduced internal rotation due to anterior impingement, reduced abduction due to acromial impingement, and prothesis dislocation due to decreased soft-tissue tension [4]. In this context the use of metallic hemi-wedges may prevent a medialized joint line and results in a lateralized baseplate and sphere position [5].

To avoid or to compensate such risks, glenoid augmentation can be achieved with bone or metal augmentation [6–10]. By both methods, a more lateralized center of rotation of the reverse prosthesis can be achieved as well as a bone preservation on the high side of the deformity. The low side of the deformity can be augmented by the wedged baseplate or the wedged bone block to allow a deformity correction in an anatomical manner.

While there is more evidence the literature regarding the degree of deformity correction, outcomes and complications of wedged BIO-RSA, there is minimal evidence reported with the deformity correction and outcome after trabecular metal augment.

The aim of this study was to determine whether a metal augment can correct the deformity towards an anatomic manner. A threshold of 10 degree of pathological glenoid version or inclination was defined to correct the deformity. Hemi-wedge glenoid base plates of 10°, 20° or 30° augments were used, which can be implanted and positioned 360° freely with regard to the orientation of the wedge depending on the deformity.

## Material and methods

### Study design and patient selection

A retrospective review of prospectively collected data was performed in 50 patients that had undergone primary RSA by the first authors (T.G., J.W. and L.N) between August 2020 and July 2022 at our institution (Orthopädische Klinik Paulinenhilfe Stuttgart) with a metal augmented base plate (Comprehensive Reverse Shoulder System Augmented Baseplate; Zimmer Biomet; Warsaw, Indiana, USA).

The following inclusion criteria were used: (1) all adults with skeletal maturity; exceeding a threshold of pathologic angular dimension of inclination in the coronal planes of > 10° (2) and version in the axial planes of > 10° (3) all RSA with a metal augmented baseplate (4) primary osteoarthritis and consented to be included in the study. Patients with pre-operative acquired or congenital acromial abnormalities such as os acromiale or stress fractures were excluded from this study.

Glenoid morphology and deformity were determined in all patients using preoperative radiographic and CT scan analysis of the affected shoulder and entire scapula, in order to classify the glenoid morphology according to the Walch [11] and Favard [12] classification. All patients received preoperative planning, CT scan (0.5-mm slices) with 3D planning (Signature One Planner; Zimmer Biomet; Warsaw, Indiana, USA).

Shoulder radiograph and CT scan were obtained for all patients at final follow-up and determined the postoperative achieved glenoid inclination (°; β angle) [13], RSA angle [12] and version (°; distal, middle, and proximal) [14]; in addition, Acromio-Humeral (AH) distance (mm), scapulo-humeral index (SCI; %) [15], were also determined. The sphere bone overhang distance (SBOD, mm)[16] was measured to estimate the scapular notching, which was rated on the anteroposterior scapular radiograph according to the system of Sirveaux et al. [17]. Radiographic studies were evaluated by two independent reviewers (experienced orthopedic surgeons), and the data were analyzed by calculating intraclass correlation coefficients (ICCs). We found excellent agreement between the two observers (ICC 0.98).

The ethics commission (Ethikkommission der Landesaerztekammer Baden-Wuerttemberg Germany, F-2022-005) approved all procedures, and the study was conducted in accordance with the Helsinki Declaration of 1975, as revised in 2008 [18].

### Surgical technique

All surgeries were performed using the Comprehensive Reverse Shoulder System (Zimmer Biomet; Warsaw, Indiana, USA). The procedure was carried out under general anesthesia with an interscalene catheter block in beach chair position. A standard deltopectoral approach was performed, with detachment of any remaining sub-scapularis and tenodesis of the long head of biceps. First, the asymmetric high side of the glenoid was reamed with a 25 mm reamer (approximately 50% of the glenoid surface) followed by a half-open second reamer of the lower sitting 50% of the bone deficient glenoid. A metal augmented glenoid base plate with a hemi-wedge of 10°, 20° or 30° was used. All base plates were placed with the maximum hemi-wedge in the zone of greatest defect, according to our preoperative planning. The sphere asymmetric guide allows for optimal offset sitting of the a 36 or 41 mm sphere at a desired offset of at least 4 mm caudal glenoid overhang. To ensure that the deltoid was appropriately tensioned and the implant was properly positioned, we (1) looked for stable prosthesis position (two fingers allowed between inlay and humeral tray) during application of axial traction on the arm, (2) ensured stability throughout a full range of motion, and (3) palpated for tension in the conjoint tendon and deltoid muscle after trial reduction [1]. The humeral stem was cementless and in < 90% a short stem, with a total neck-shaft angle of 147.5°.

### Statistical analysis

Descriptive statistics, including means and standard deviations, were reported for demographic data and outcome variables. Comparisons between different measurements had a normal distribution and therefore were analyzed for significance using the unpaired Student *t* test. The level of statistical significance was set at *p* < 0.05. Statistical analysis was performed with SPSS 18.0 software (SPSS Inc., Chicago, IL, USA).

### Source of funding

A funding from Zimmer Biomet was applied to follow-up patients.

## Results

A total of 48 patients (50 shoulders) who had undergone a reverse shoulder arthroplasty with a metal augmented glenoid met our inclusion criteria and were enrolled in the study. The majority of patients were male (*n* = 26; 52%). The mean age was 69 ± 12.65 (42–90) years.

The average postoperative follow-up was 6 months (1–23 months) with a minimum of 1-month follow-up. Patient demographics, pre-operative diagnosis and glenoid morphology (Walch and Favard classification) are presented in Table 1.

**Table 1:** Patient demographic data

	Value
Total no. of patients	48 (50 shoulders)
Side, *n* (%)	
Right	36 (72%)
Left	14 (28%)
Gender, *n* (%)	
Male	26 (52%)
Female	24 (48%)

There were no scapular stress fractures in our study group during short term follow up.

### Individual correction of each glenoid deformity

In the majority of cases the wedge was positioned posteriorly and cranially between 10:00 and 12:00 o’clock. A total of 24 (48%) 10° wedges, 18 (36%) 20° wedges and 8 (16%) 30° wedges were used.

For a better understanding of the individual correction of each deformity, we have divided the total study population of patients with deformities into three groups.

#### Patients presenting with global deformity (total study population, group I)

Preoperative and postoperative correction data for all patients 48 patients (50 shoulders) are presented in Table 2.

**Table 2:** Preoperative and postoperative data from all patients

Data	Preoperative	Postoperative	*p* value
RSA angle	19.8 ± 7.37 (2.3 to 32.7)	0.56 ± 2.89 (− 5.6 to 5.5)	< 0.0001
Glenoid version (distal)	− 6.61 ± 8.11 (− 24.1 to 10.2)	− 8.23 ± 7.47 (− 26.1 to 2.2)	0.1507
Glenoid version (middle)	− 9.41 ± 9.19 (− 24.6 to 6.7)	− 7.86 ± 5.75 (− 20.4 to 2.2)	0.1512
Glenoid version (proximal)	− 11.7 ± 11.44 (− 32.1 to 14.4)	− 7.46 ± 5.92 (− 20.4 to 2.5)	0.0117
SCI	60 ± 8 (44 to 77)	50	< 0.0001
AH—distance	6.29 ± 4.07 (0.1 to 13.5)		
SBOD		5.32 ± 1.99 (2.6 to 10.8)	
Glenoid morphology			
Walch Type B1	20 (40%)		
Walch Type B2	25 (50%)		
Walch Type D	5 (10%)		
Favard Type E0	13 (26%)		
Favard Type E1	26 (52%)		
Favard Type E2	3 (6%)		
Favard Type E3	8 (16%)		

Glenoid inclination—β angle (°); Reversed Shoulder (RSA) angle (°); Glenoid Version (°); Acromio-Humeral (AH) distance (mm); scapulo-humeral index (SCI; %); Sphere bone overhang distance (SBOD, mm)

Scapular notching was not observed in our patients and relevant perioperative complications were not observed.

#### Patients with a predominantly glenoid retroversion (> 20°; group 2)

Preoperative and postoperative correction data for 20 patients (20 shoulders) with a glenoid retroversion greater than 20° are presented in Table 3.

**Table 3:** Preoperative and postoperative data

Data	Preoperative	Postoperative	*p* value
RSA angle	15.91 ± 6.55 (2.3 to 25.8)	0.08 ± 3.45 (− 5.6 to 5.5)	< 0.0001
Glenoid version (distal)	− 13.62 ± 4.25 (− 24.1 to − 5.7)	− 7.90 ± 7.321 (− 20 to 2.2)	0.0095
Glenoid version (middle)	− 18.93 ± 3.35 (− 24.6 to − 15)	− 7.66 ± 5.28 (− 16 to − 1.8)	< 0.0001
Glenoid version (proximal)	− 23.32 ± 4.56 (− 32.1 to − 12)	− 6.74 ± 5.75 (− 16.5 to 0.2)	< 0.0001
SCI	64 ± 7 (56 to 77)	50	< 0.0001
AH—distance	6.37 ± 3.74 (1.2 to 12.4)		
SBOD		5.70 ± 2.04 (2.6 to 10.8)	
Glenoid morphology			
Walch Type B1	6 (30%)		
Walch Type B2	14 (70%)		
Favard Type E0	9 (45%)		
Favard Type E1	11 (55%)		

Glenoid inclination—β angle (°); Reversed Shoulder (RSA) angle (°); Glenoid Version (°); Acromio-Humeral (AH) distance (mm); scapulo-humeral index (SCI; %); sphere bone overhang distance (SBOD, mm)

The number of female and male patients was equal. The mean age was 67 ± 12 (42–86) years. The predominant side operated was right (12 (60%)). In the majority of cases the wedge was positioned posteriorly and cranially at 10:30 o’clock. A total of 7 (35%) 10° wedges, 6 (30%) 20° wedges and 7 (35%) 30° wedges were used.

Scapular notching was not observed in our patients and relevant perioperative complications were not observed.

**Fig. 1:**
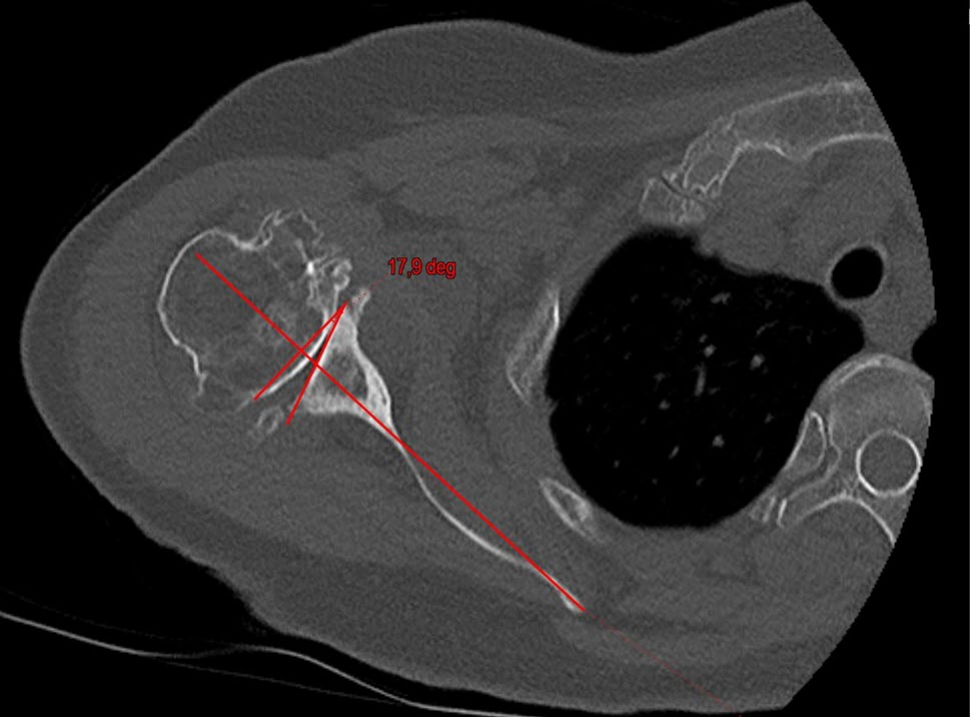
Preoperative glenoid retroversion

**Fig. 2:**
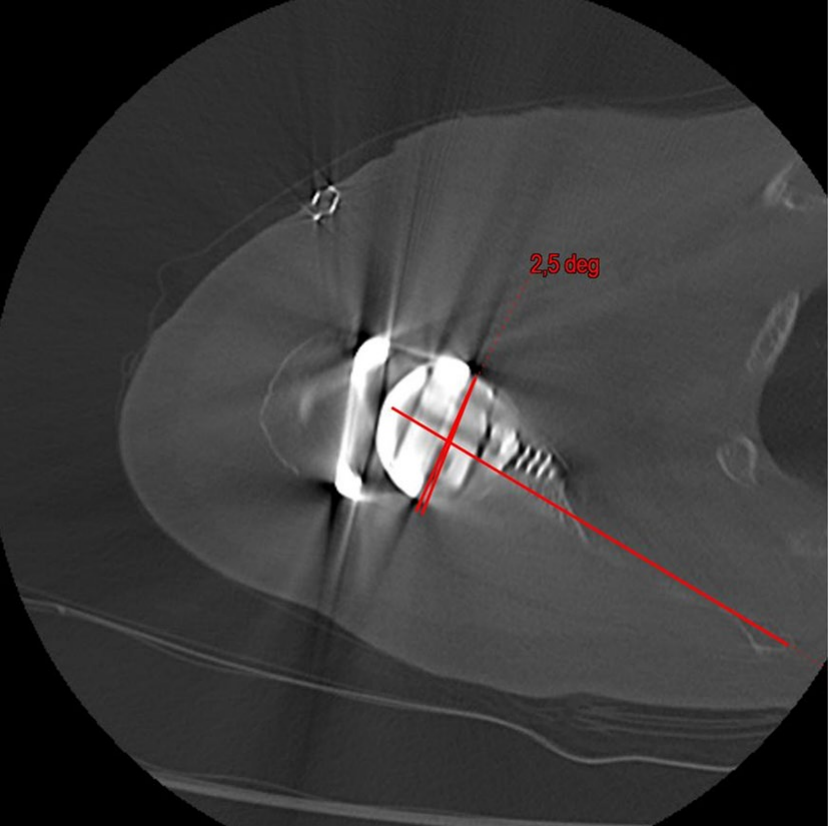
Postoperative glenoid retroversion

**Fig. 3:**
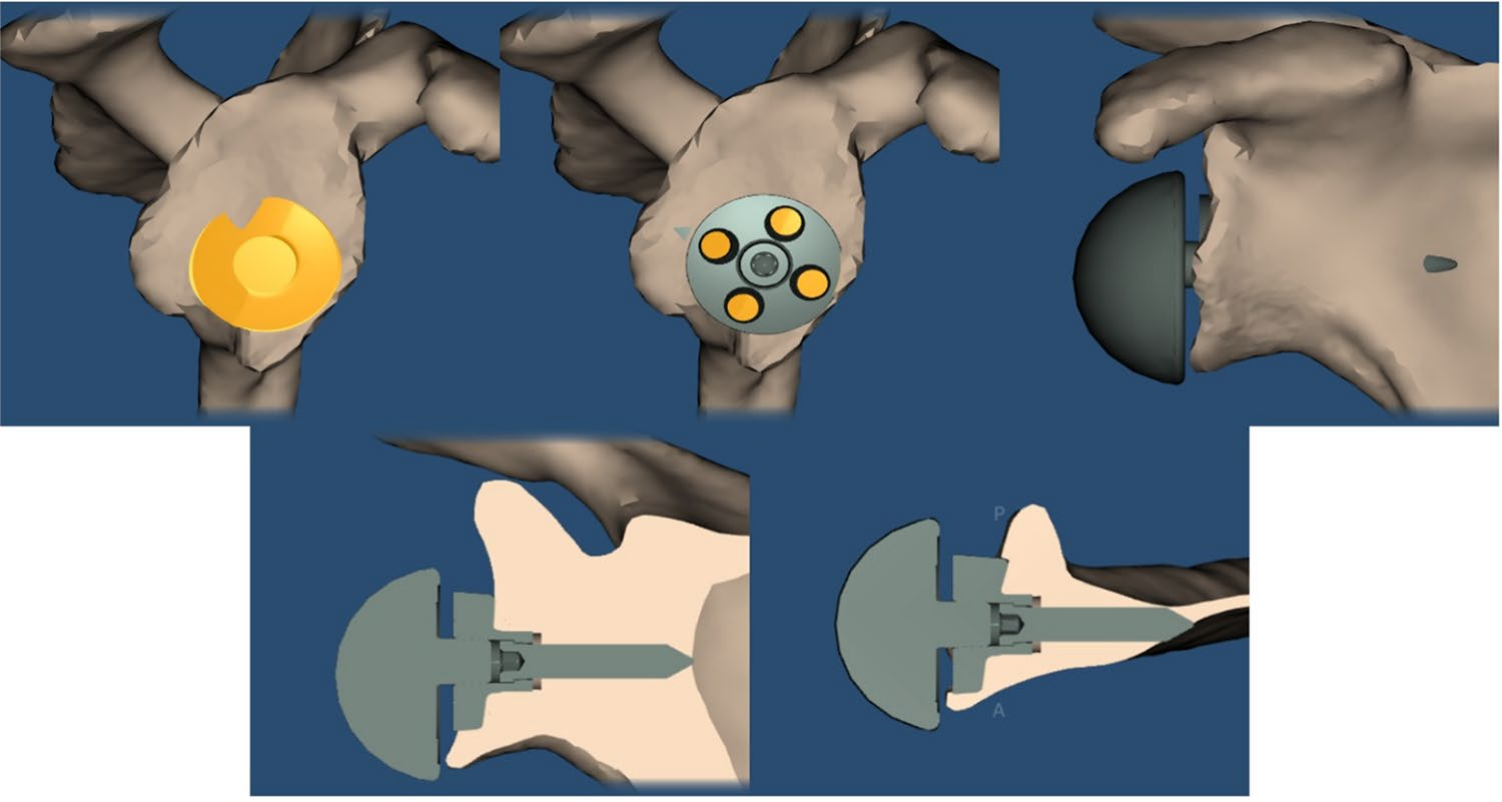
Preoperative planning image using Signature One Planner®

Figures 1 and 2 show the preoperative and postoperative retroversion. Figure 3 shows the preoperative planning image using Signature One Planner.

#### Patients with a predominantly cranial glenoid dysplasia (group III)

Preoperative and postoperative correction data for 17 patients (17 shoulders) with a cranial glenoid dysplasia are presented in Table 4.

**Table 4:** Preoperative and postoperative data

Data	Preoperative	Postoperative	*p* value
RSA angle	22.76 ± 6.06 (12.1 to 32.7)	0.19 ± 2.7 (− 5.6 to 5.1)	< 0.0001
Glenoid version (distal)	− 3.72 ± 7.63 (− 16.2 to 10.2)	− 6.5 ± 6.53 (− 26.1 to 1.9)	0.1704
Glenoid version (middle)	− 5.83 ± 9.32 (− 24.6 to 6.7)	− 6.93 ± 4.96 (− 20.4 to − 1.8)	0.3693
Glenoid version (proximal)	− 7.55 ± 10.82 (− 27.6 to 14.4)	− 7.39 ± 4.33 (− 20.4 to 2)	0.4630
SCI	57 ± 9 (44 to 77)	50	0.0055
AH – distance	5.08 ± 4.2 (0.1 to 1.8)		
SBOD		5.69 ± 1.88 (2.9 to 10.8)	
Glenoid morphology			
Walch Type B1	8 (47%)		
Walch Type B2	9 (53%)		
Favard Type E1	12 (71%)		
Favard Type E3	5 (29%)		

Glenoid inclination—β angle (°); Reversed Shoulder (RSA) angle (°); Glenoid Version (°); Acromio-Humeral (AH) distance (mm); scapulo-humeral index (SCI; %); Sphere bone overhang distance (SBOD, mm)

The majority of patients were female (*n* = 10; 59%). The mean age was 71 ± 13 (42–90) years. The predominant side operated was right (12 (70.59%)). In all cases the wedge was positioned cranially at 12:00 o'clock to correct the inclination. A total of 12 (70.59%) 10° wedges and 5 (29.41%) 20° wedges were used.

Relevant perioperative complications were not observed.

**Fig. 4:**
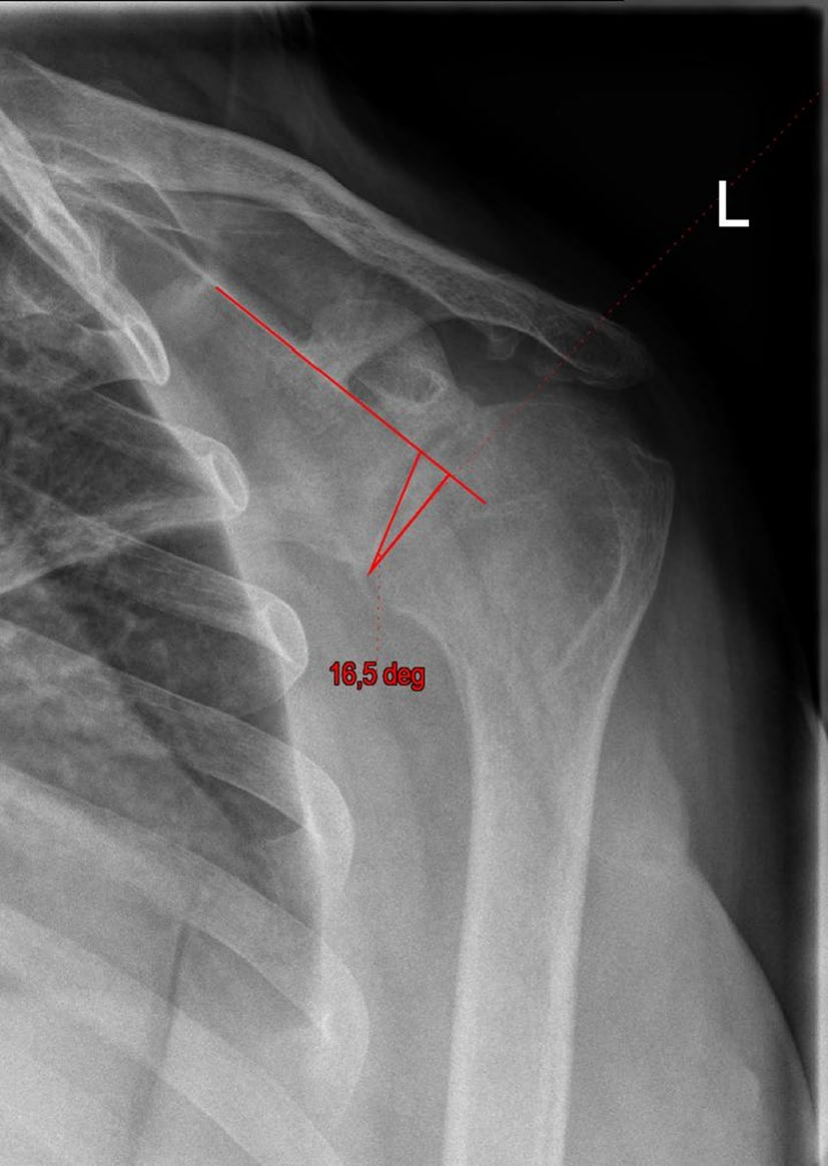
Preoperative RSA angle

**Fig. 5:**
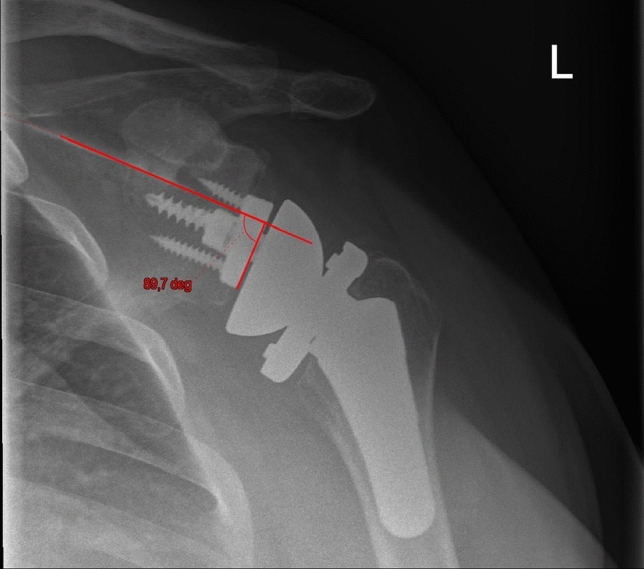
Postoperative RSA angle after using a 10° augment

**Fig. 6:**
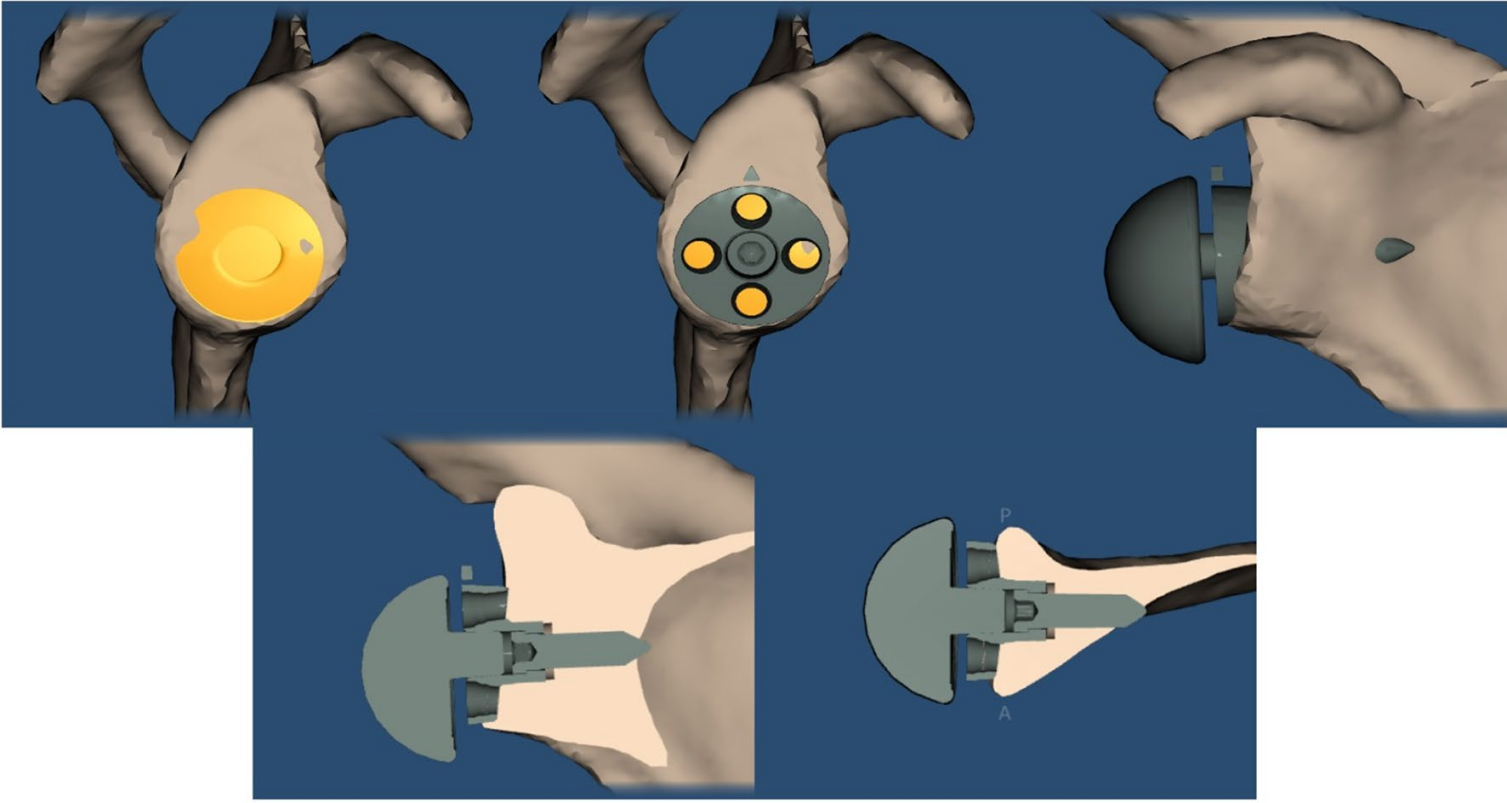
Preoperative planning image using Signature One Planner®

**Fig. 7:**
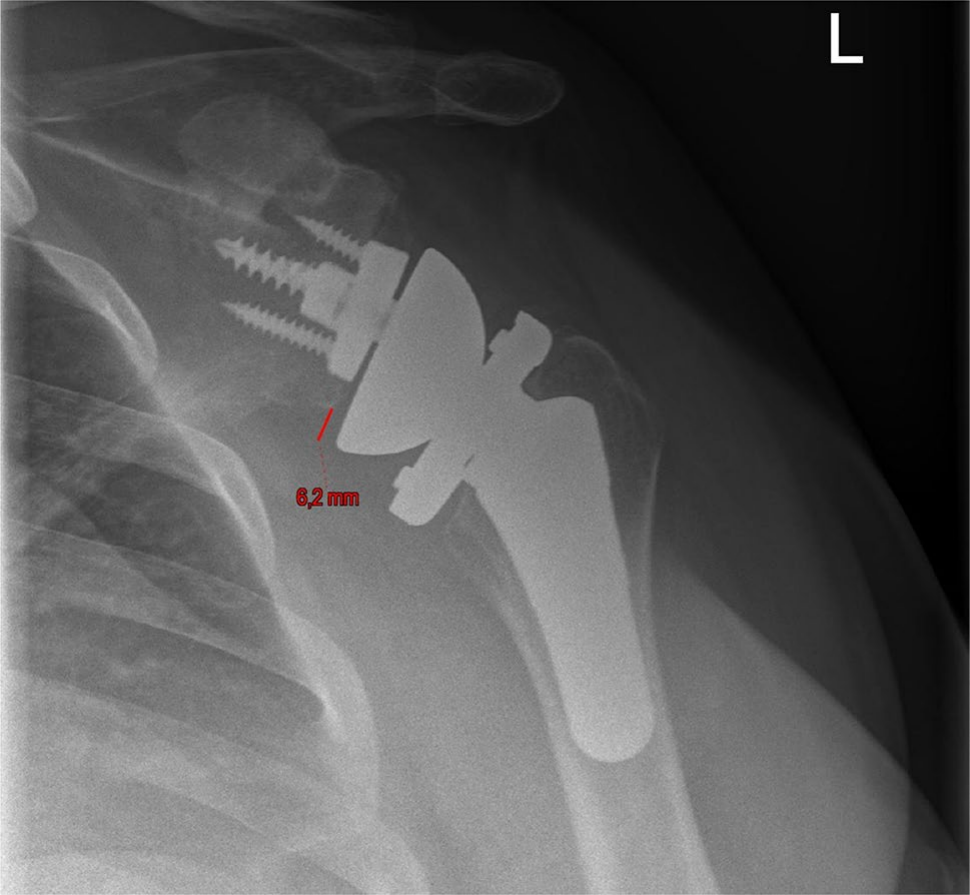
Postoperative SBOD (Sphere Bone Overhang Distance)

Figures 4 and 5 show the preoperative and postoperative RSA angle. Figure 6 shows the preoperative planning image using Signature One Planner. Figure 7 shows the postoperative SBOD.

## Discussion

The objective of the current study was to analyze pre- and postoperative shoulder radiographs and CT scans to determine the correction of a glenoid deformity in RSA using a metal augmented hemi-wedge baseplate. We measured pre- and postoperative glenoid version, inclination, distalisation and lateralization to evaluate the 360-degree correction potential of the preoperative deformity.

Preoperatively, we classified the glenoid deformity according to the Walch [11] and Favard classifications [4, 13]. While the optimum method for correcting superior glenoid inclination in RSA is debatable, it is crucial to rectify it to a neutral position (RSA angle 0°) and thereby avoid the superior baseplate tilt [19, 20]. For the measurement of the glenoid inclination (RSA angle), the supraspinatus fossa line is a consistent reference line due to this sclerotic line is visible on true AP shoulder radiographs and CT scans. In addition, from a biomechanical perspective, the supraspinatus fossa line points the course of action of the rotator cuff muscles [21]. The goal of preoperative planning in RSA should therefore be to achieve an RSA angle measurement of 0°, which means that the baseplate should be implanted in neutral inclination in relation to the supraspinatus fossa line. In such a scenario, the trajectories of the other cuff muscles are orthogonal and potentially more efficient [12, 21–23].

Various techniques have been proposed to correct the superior glenoid inclination by performing a RSA [20, 24]. A 2016 Nebraska study demonstrated that neither the subchondral smile reaming technique nor the 10° cannulated guide pin technique are reliable methods for correcting superior glenoid tilt [25].

Possibly not all glenoid defects may require deformity correction. This is supported by reports from McFarland et al. Here a medialized center of rotation with asymmetric reaming was chosen for best possible baseplate positioning, which however does not necessarily lead to a sufficient deformity correction and may lead to a violation of the subchondral bone [3]. However, in order to correct a large amount of superior inclination or retroversion, it may require reaming out a large amount of native bone, potentially endangering the bone mass and too much medializing the glenoid component, as well as the center of rotation, which can lead to prosthetic instability, scapular notching, glenoid loosening and reduced mobility [8, 26, 27]. To prevent this, there are three reliable surgical options that can be used: (1) inferiorly inclined bone graft as wedged BIO-RSA [27], (2) patient-specific baseplate or (3) metal superior augmented baseplate [5].

Therefore, in our surgical practice, our preference is the use of a metal augmented hemi-wedge baseplate once a threshold of 10° of pathological glenoid version or inclination is exceeded. This hemi-wedge baseplate allows a correction in at least 2 planes in combination with a bone preserving reaming-technique. In particular, the convenience of this technique combines the adaptability to individually reconstruct defects not only of the glenoid inclination but also of the version, as well as the better adaptation of the baseplate without graft compression like in the BIO-RSA [27–29]. In our hands this technique is not time-consuming in the OR after a learning curve, nor it is associated with extensively higher implant expense, and does avoid the need for a bone integration of a wedged bone block.

To correct preoperative deformity, a 3D planning system is mandatory, to allow a perfect positioning of the augmented baseplate. We use the Signature™ ONE surgical planning software[30] (Zimmer Biomet, Warsaw, Indiana, USA) which provides a 3D image-based approach to preoperative visualization, surgical planning and patient-specific guide creation, based on each patient´s unique anatomy, to anticipate the direction and size of the metallic augmentation.

The results show that the mean preoperative RSA angle in our total cohort in our study was 19.8° ± 7.37 (2.3–32.7), which clearly shows an increased superior inclination. The mean preoperative results of the glenoid version calculation demonstrate that most of the glenoids were in retroversion. The highest retroversion of the glenoid is evidenced in the proximal section (− 11.7 ± 11.44 (− 32.1 ± 14.4)), in accordance with prevision measurements [31]. Anatomically the reduction of the retroversion at the middle of the glenoid is the demonstration of the spiral twist of the glenoid (− 9.41 ± 9.19 (− 24.6 to 6.7)), and so on at the distal section (− 6.61 ± 8.11 (− 24.1 to 10.2)). Postoperative after the implantation of a metallic augmented hemi-wedge basis plate we were able, first, to anatomically correct the RSA angle to 0.56° ± 2.89 (− 5.6 to 5.5) and, second, the glenoid retroversion in the proximal section to − 7.46 ± 5.92 (− 20.4 to 2.5), as well as in the middle section to − 7.86 ± 5.75 (− 20.4 to 2.2). This is due to the fact that our metallic augmented basis plates were positioned between 10 and 12 o’clock; we can demonstrate that it has been possible to make a 3D correction particularly in the proximal and middle glenoid section.

The results further demonstrate that in our group with a retroversion greater than 20° at the proximal third we were able to correct combined both the retroversion as well as the inclination in that we positioned the metallic augment posteriorly and cranially (10:30 o’clock). There was a statistical significance (*p* < 0.05) in the correction of these deformity on the RSA angle from 16°, the glenoid version at the proximal third from 23°, at the middle third from 19° and at the distal third from 13.81 to 0.08°, − 7.87°, − 7.03° and 7.98°, respectively. These data clearly demonstrate that by adjustment of the wedge at the 10:30 o’clock, we were able to correct the version to the largest degree on the upper third of the glenoid, rather than on the distal part of the glenoid. In our group 3, in patients with a cranial glenoid dysplasia we were able to statistical significant (*p* < 0.0001) reconstruct the RSA angle from 22.76° ± 6.06 (12.1–32.7) to 0.19° ± 2.7 (− 5.6 to 5.1). In this group were predominantly a dysplasia Favard Type E1 which with its concentric erosion presents a risk of a superior baseplate tilt and therefore a challenge to achieve an anatomically reconstruction. Because of the positioning of the metallic augmented base plate at 12:00 o’clock, the glenoid version was not affected by the augment.

Another important aspect by the glenoid positioning is attempted to avoid a scapular notching by both lateralization and by inferiorizing the glenoid base plate. Notching is hypothesized to be secondary to the contact between the polyethylene on lay tray and the bone just inferior and medial to the base plate. Further bone loss occurs as a result of this direct contact and results in polyethylene debris particles that can lead to additional bone loss and possible aseptic loosening of the glenoid or humeral component [17, 32–35].

A recent study from the Mayo clinic showed that a SBOD below 2.35 mm is a strong predictor for a greater notching grade [16]. In our study, scapular notching was not observed, what is strongly supported by our postoperative SBOD of 5.32 mm ± 1.99 (2.6–10.8).

There are few more reports of correction of glenoid dysplasia by metal augments or wedged BIO-RSA. Boileau et al. reported an considerably improvement of the RSA angle from preoperative numbers of 28° to 8.6°, and an improvement of the glenoid version from − 12.1° to − 4.7° postoperatively after wedged BIO-RSA [8]. Similarly, Kirsch et al. reported an improved version from 28° to 16° after usage of an metallic implant, and an improved β-angle from 67° to 81° postoperatively, respectively [36].

In the last year there has been increasing interest in metal augments in order to correct deformities and to prevent medialization of the joint line. Zhou et al. described very recently the use of metallic hemi-wedges similar to ours and showed that they were able to prevent a medialized joint line resulting in a lateralized baseplate and sphere position [5].

Eventually, these newer bone preserving techniques including the usage of the presented hemi-wedge need to be compared with the good mid-term results after usage of a standard baseplate without bone grafting or augments as proposed [3]. Therefore, it is mandatory to collect such long term clinical and radiographic data to evaluate the benefits of deformity correction by bone and metal augments compared to current standard techniques without correction.

### Limitations

First, our patient population includes only patients with Osteoarthrosis. However, in our database there were some fractures or the proximal humerus, which were indicated to RSA. Second, our study was only based on radiographic images, and in consequence it does not include patient-reported outcome measures. Therefore, the final valuation whether metallic augments are beneficial is dependent on the clinical long-term outcome and revision rates. Third, our metallic augmented base plate hat fixed 10°, 20° or 30, which limited our judgement to more closely achieve an anatomical result. The current study results were not able to show the degree of bone preservation by the use of a wedged glenoid. We need to address this important aspect in further volumetric measurements.

## Conclusion

Using a new 360-degree metal-augmented hemi-baseplate, the preoperative pathological inclination and retroversion can be corrected in a combined and/or individually reliable way. Future clinical long-term results will show whether this will lead to even improved clinical outcomes compared to wedged BIO-RSA or standard baseplates without deformity correction.

## Data Availability

The datasets used and/or analyzed during the current study are available from the corresponding author on reasonable request.
